# Syndrome de Sturge Weber Krabbe: entité exceptionnelle (à propos d'un cas)

**DOI:** 10.11604/pamj.2018.31.211.14606

**Published:** 2018-11-28

**Authors:** Siham Alaoui Rachidi, Anas Lahlou Mimi, Amal Akammar, Youssef Lamrani Alaoui, Meriem Boubbou, Mustapha Maaroufi, Badr Alami

**Affiliations:** 1 Service de Radiologie, CHU Hassan II-Fès, Rabat, Maroc

**Keywords:** Angiome, IRM cérébrale, enfant, Sturge Weber, Angioma, brain MRI, child, Sturge Weber

## Abstract

Nous rapportons le cas d'un syndrome de Sturge Weber diagnostiqué au sein du Service de Radiologie de CHU Hassan II de Fès. A partir de cette observation, nous montrons les aspects cliniques, diagnostiques et thérapeutiques ainsi qu'évolutifs de cette entité neuro radiologique exceptionnelle.

## Abstract (en)

We report a case of Sturge-Weber-Krabbe Syndrome diagnosed in the Department of Radiology at the Hassan II University Hospital in Fes. This study highlights the clinical diagnostic and therapeutic features as well as the evolutionary characteristics of this uncommon neuroradiologic disorder.

## Introduction

Le syndrome de Sturge-Weber-Krabbe ou angiomatose encéphalo-trijéminée associe un angiome facial congénital, un angiome leptoméningé et angiome choroïdien [1]. C'est une affection sporadique, touchant les deux sexes avec une discrète prédominance masculine [1]. Elle reste une maladie très rare sur l'échelle mondiale et surtout au Maroc, seulement quelques cas ont été rapportés jusqu'à maintenant. Cliniquement, le mode de révélation est l'épilepsie dans 75 à 90% des cas [1]. L'IRM reste l'examen de choix dans le dépistage précoce et le suivi de cette pathologie [1, 2].

## Patient et observation

**Figure 1:**
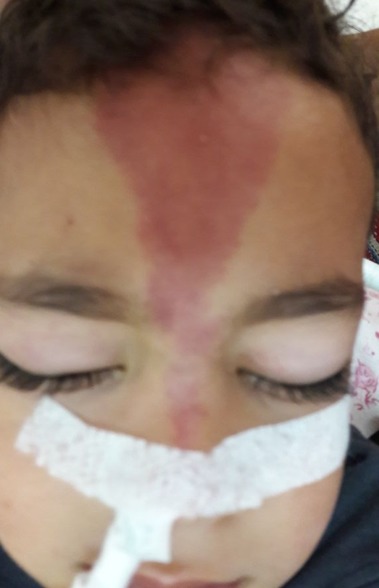
angiome facial du territoire du V1

**Figure 2:**
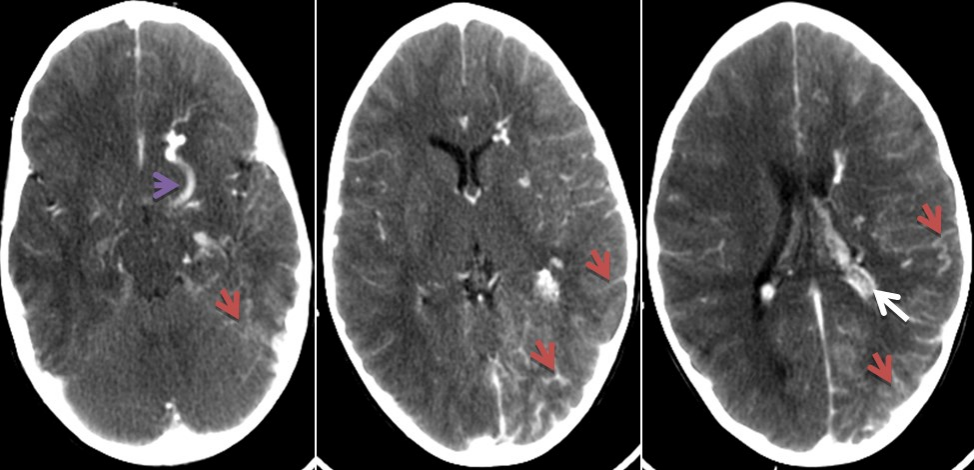
TDM cérébrale après injection de produit de contraste: rehaussement méningo-pial hémisphérique gauche (flèche rouge) associé à une hypertrophie du plexus choroïde homolatéral (flèche blanche) ainsi qu'une dilatation des veines trans-cérébrales (flèche mauve)

**Figure 3:**
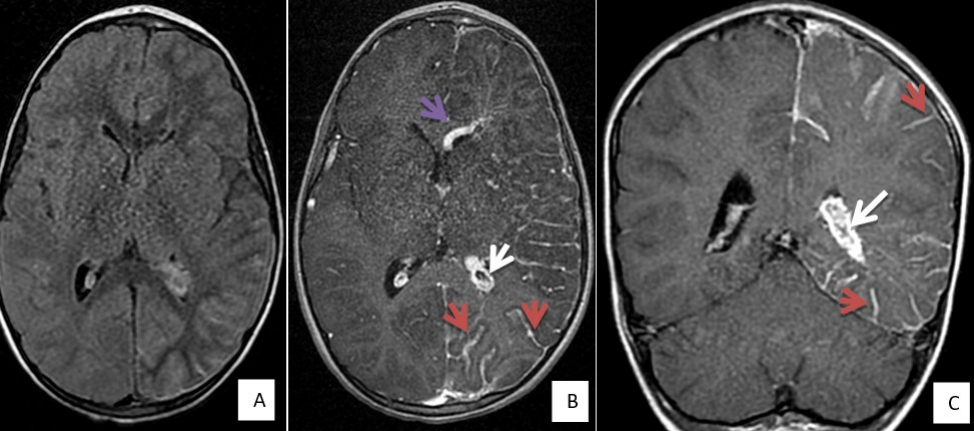
IRM cérébrale avec une séquence axial Flair (A), axial T1 C+ (B) et coronale T1 C+ (C): objectivant les mêmes anomalies sus décrites en TDM avec un rehaussement méningo-pial plus prononcé au niveau hémisphérique gauche (flèche rouge) associé à une hypertrophie du plexus choroïde homolatéral (flèche blanche) ainsi qu'une dilatation des veines trans-cérébrales (flèche mauve)

Ayman M, enfant de 03 ans et 07 mois a été admis le 17/07/17 pour état de mal épileptique. On n'a pas noté d'épilepsie aux ATCD personnelle ou familiale, pas de notion de consanguinité, et l'enfant avait jusqu'à ce jour un bon développement psychomoteur. Le début de la symptomatologie était brutal par l'installation le jour même des crises convulsives rapprochées, sans notion de prise de conscience entre les crises, le tout évoluant dans un contexte de fièvre, ce qui a motivé la famille a consulté en urgence dans notre formation pour prise en charge. A l'examen, Ayman était inconscient, fébrile à 39°C, sans déficit sensitivo moteur, et au reste de l'examen on a objectivé un angiome plan cutané au niveau de la face occupant le territoire du V1 (Figure 1). L'enfant a été intubé en urgence sur des critères neurologiques. Une TDM cérébrale a été réalisée par la suite objectivant un angiome Méningo-pial gauche associé à une hypertrophie du plexus choroïde homolatéral ainsi qu'une dilatation des veines transcérébrales (Figure 2). Une IRM fut pratiquée secondairement objectivant la présence de quelques structures vasculaires serpigineuses frontales gauches avec une dilatation du plexus choroïde homolatéral ainsi qu'une prise de contraste accentuée des sillons corticaux hémisphériques gauches, qui sont discrètement élargies rentrant dans le cadre de sa malformation angiomateuse (Figure 3). A l'EEG, on a mis en évidence un foyer de souffrance pariéto-occipitale gauche et l'examen ophtalmologique était normal. Au total, le diagnostic du syndrome de Sturge Weber a été retenu, l'enfant a été mis sous traitement médical à base d'anti-épileptique avec une bonne amélioration clinique immédiate (reprise de conscience et arrêt de crises convulsives).

## Discussion

Le syndrome de Sturge-Weber Krabbe est une phacomatose neuro-cutanée et oculaire à substratum malformatif vasculaire très rare, dont l'atteinte cutanée est souvent unilatérale atteignant le territoire du nerf trijumeau. Sa présence est très évocatrice du diagnostic mais son absence ne l'exclut pas, les atteintes oculaires sont dominées par le glaucome (30-70%), un angiome choroïdien est retrouvé dans 40-50% des cas, le fond d'œil recherchera alors un soulèvement rétinien rougeâtre [1, 3, 4]. Les signes neurologiques sont dominés par l'épilepsie (75 à 90% des cas), souvent précoce et sévère, des crises motrices partielles de l'hémicorps controlatéral dans 70% des cas. Le déficit moteur ainsi que le retard mental sont retrouvés dans 50% des cas. Des troubles psychiatriques, qui ont été décrites, restent rares [1, 5]. Une classification a été proposée par Roch et Coll. qui décrit 3 formes de Sturge Weber: type 1 (classique): manifestations intracrâniennes et faciales; Type 2: atteinte faciale seule sans modifications centrales; Type 3: manifestations intracrâniennes seules. Notre cas appartient au syndrome de Sturge Weber type 1 selon Roch et Coll. Le principal symptôme constaté chez notre malade correspond à des crises convulsives compliquées d'état de mal épileptique, sans déficit ni retard mental. L'angiome cutané facial dans le territoire du V1 avec sa couleur en lie de vin, nous a beaucoup aidé dans la suspicion du diagnostic avant même la réalisation de l'imagerie. L'imagerie en coupes joue un rôle primordial dans le diagnostic du syndrome de Sturge Weber. L'IRM est l'examen de choix dans le dépistage précoce et le suivi de cette pathologie [1, 6]. La TDM cérébrale recherchera: une atrophie cérébrale, focale ou hémisphérique, souvent homolatérale à l'angiome; des calcifications intra crâniennes en forme de « S », gyriformes ou en rail de train, de siège sous corticales, au niveau des artères méningées et des veines corticales; une hypertrophie et calcifications du plexus choroïde homolatéral à l'angiome; une prise de contraste corticale, gyriforme.

L'IRM cérébrale, plus sensible que le scanner [6], permet d'objectiver: les signes précoces même avant la clinique; l'angiome pie-mérien: intérêt des séquences d'ARM++; l'angiome du plexus choroïde; les anomalies de développement veineux; l'atrophie cérébrale; les calcifications cérébrales en hypo signal sur toutes les séquences: séquences écho de gradient (EG) pondérées en T2 +++; une polymicrogyrie, une lissencéphalie ou une pachygyrie localisée. L'angiographie n'est plus pratiquée sauf en cas de maladie épileptique grave, où une hémisphérectomie palliative est proposée, son intérêt est d'évaluer mieux l'extension de l'angiome [6]. L'imagerie en coupe (TDM et IRM) chez notre patient rejoint les signes fréquemment retrouvés en littérature, avec un rehaussement méningo-pial, dilatation des veines cérébrales profondes, hypertrophie du plexus choroïde du même côté, les calcifications n'étaient pas présentes dans notre cas vu le diagnostic précoce de la maladie. L'imagerie cérébrale fonctionnelle n'est pas de pratique courante, elle présente des indications particulières, permettant souvent un diagnostic précoce, en étudiant le métabolisme cérébral du glucose par la tomographie à positon (PET) et celle du débit sanguin cérébral régional par imagerie fonctionnelle type Single photon EmissionTomography(SPECT) [7, 8], ces examens rechercheront: à un stade précoce de la maladie: un hyper-métabolisme régional transitoire du cortex au niveau de l'angiome pial; à une phase avancée: un hypo-métabolismeen PET et une hypo-perfusion en SPECT au niveau des zones calcifiées. L'EEG est souvent anormal montrant un ralentissement de l'activité de fond dans un ou les deux hémisphères en rapport avec une souffrance cérébrale [1, 9], comme l'a confirmé notre cas. Le traitement est polyvalent, préventif et curatif, basé sur les anti-épileptiques. Quant au traitement chirurgical de l'angiome pial, il consiste en une hémisphérectomie et doit être envisagé pour les formes unilatérales d'évolution sévère et grave de l'épilepsie ou de régression intellectuelle [10, 11]. Pour le glaucome, le traitement médical est souvent préféré vu les risques de complications per et post-opératoires non négligeables, mais souvent inefficaces [11, 12]. Notre malade a bénéficié seulement d'antiépileptique, avec une bonne amélioration clinique, il n'avait pas besoin d'un traitement chirurgical.

## Conclusion

Le diagnostic de Sturge Weber repose sur la constatation d'un angiome facial plan, atteignant au moins le territoire de la première branche du trijumeau et d'un angiome hémisphérique cortico-pie-mérien homolatéral. Il repose avant tout sur l'imagerie cérébrale moderne, IRM ou TDM avec injection de produit de contraste.

## Conflits d’intérêts

Les auteurs ne déclarent aucun conflit d’intérêts.
